# Changes in Nucleolin Expression during Malignant Transformation Leading to Ovarian High-Grade Serous Carcinoma

**DOI:** 10.3390/cancers15030661

**Published:** 2023-01-21

**Authors:** Elizabeth A. Paris, Janice M. Bahr, Sanjib Basu, Animesh Barua

**Affiliations:** 1 Department of Anatomy & Cell Biology, Rush University Medical Center, Chicago, IL 60612, USA; 2 Department of Animal Sciences, University of Illinois at Urbana-Champaign, Urbana, IL 61801, USA; 3 Department of Internal Medicine, Rush University Medical Center, Chicago, IL 60612, USA; 4 Department of Pathology, Rush University Medical Center, Chicago, IL 60612, USA; 5 Department of Obstetrics and Gynecology, Rush University Medical Center, Chicago, IL 60612, USA

**Keywords:** ovarian cancer, early detection, nucleolin, serum marker, fimbria, chicken model, ultrasound imaging

## Abstract

**Simple Summary:**

Ovarian cancer (OVCA) is one of the most fatal gynecologic malignancies with most cases detected in late stages. High mortality rates are due, in part, to the lack of an effective early detection test. Tumor development and progression are associated with an increase in the expression of tissue proteins and/or their release into circulation. These proteins may offer potential markers for the early detection of OVCA. Nucleolin, a nuclear protein, is one such marker. This pilot study examined whether the tissue expression of nucleolin and its serum levels change in association with OVCA development and progression by using archived clinical samples and the laying hen model of spontaneous OVCA. Nucleolin was found to be highly expressed in ovaries and fimbriae with tumors and in serum levels compared to those without OVCA. This study also found that tumor-associated serum levels of nucleolin increase even before the tumor forms a detectable solid mass in the ovary or fimbria, suggesting that nucleolin may be involved in malignant transformation. A smaller sample size is a limitation of this study; however, it will lay the foundation for clinical studies with larger cohorts to examine the efficacy of nucleolin as a potential serum marker for the early detection of OVCA.

**Abstract:**

Objective: Ovarian high-grade serous carcinoma (HGSC) is a fatal malignancy of women. Alterations in the expression of nuclear proteins are early steps in malignant transformation; nucleolin is one such protein. Changes in nucleolin expression and circulatory levels during ovarian HGSC development are unknown. The study goal was to determine if tissue and circulatory levels of nucleolin change in response to malignant transformation leading to ovarian HGSC. Methods: Sera, ovaries, and BRCA+ fimbria from healthy subjects, and sera and tumor tissues from patients (*n* = 10 each), and healthy hens and hens with HGSC were examined in exploratory and prospective studies for nucleolin expression by immunohistochemistry, immunoblotting, gene expression, and immunoassay, and analyzed by analysis of variance (ANOVA). Results: Compared with normal, nucleolin expression was higher in patients and hens with ovarian HGSC and in women with a risk of HGSC (*P* < 0.05). Compared with normal (1400 + 105 pg/mL, *n* = 8), serum nucleolin levels were 1.5 and 1.7-fold higher in patients with early- (*n* = 5) and late-stage (*n* = 5) HGSC, respectively. Additionally, serum nucleolin levels increased significantly (*P* < 0.05) prior to the formation of detectable masses. Conclusion: This pilot study concluded that tissue and serum levels of nucleolin increase in association with malignant changes in ovaries and fimbriae leading to ovarian HGSC.

## 1. Introduction

Ovarian cancer (OVCA) is a fatal gynecological malignancy with heterogeneous origin. High-grade serous carcinoma (HGSC), the most prevalent and aggressive form of OVCA, may originate in the ovary or the fimbria of the fallopian tube [1]. Ovarian HGSC when detected in early FIGO (International Federation of Gynecology and Obstetrics) stages yields a 75–90% 5-year survival rate as opposed to around 30% 5-year survival rate when detected in late stages [2,3]. Unfortunately, the majority of cases are detected in late stages due, in part, to the lack of an effective early detection test [4]. Serum levels of CA-125 with or without transvaginal ultrasound (TVUS) imaging are the currently used method(s) for the detection of ovarian abnormalities including malignancy; CA-125 levels show only a moderate specificity and sensitivity for early-stage OVCA [5,6]. Thus, an effective test for the detection of early and/or pre-metastatic changes in ovarian HGSC is crucial for improving the survival rates of OVCA patients.

Cell nuclei are sites of origin of malignant transformation and have been used to diagnose malignancy. During malignant transformation, the nucleus of the cell undergoes profound changes including increasing in size and becoming irregular in shape [7]. The expression of nuclear proteins consequently changes with alterations in nuclear organization. This may lead to the shedding of nuclear proteins into circulation [8]. Nucleolin is a nuclear matrix protein found in the nucleolus, which is associated with the maintenance of the normal structure and function of the nucleus, among other cellular processes [9].

Although no mutation or splicing variants of nucleolin have been found to be associated with malignancy, a deregulated accumulation of nucleolin mRNA and/or protein has been reported in several cancers [10,11,12,13,14]. The Human Protein Atlas additionally details that aberrant nucleolin expression is present in multiple cancers, including ovarian cancer [15,16]. However, it is unknown whether enhanced expression of nucleolin is a consequence of malignant development, or it participates in malignant transformation and/or progression. Moreover, it is also unknown if the expression of nucleolin changes in tissues with a risk of OVCA development (premalignant change) or whether the levels of nucleolin in serum increase during malignant transformation, development, and progression of OVCA.

Difficulties in identifying patients with early-stage OVCA and access to their specimens are significant barriers to studying the involvement of nucleolin in early ovarian malignant transformation. Laying hens have been shown to develop OVCA spontaneously with remarkable similarities to OVCA in women, including histopathology, mode of dissemination, and expression of several molecular markers [17,18,19,20]. Furthermore, as in OVCA patients, the fimbria of the oviduct in hens has been reported to be a site of origin for ovarian HGSC [21].

The goal of this study was to examine whether the expression of nucleolin changes during ovarian HGSC development and progression as well as to examine whether nucleolin is shed into circulation in association with OVCA development. Since access to patients with premalignant and early-stage OVCA is difficult, a prospective part of the study was conducted in laying hens, a model for spontaneous OVCA.

## 2. Materials and Methods

### 2.1. Clinical Specimens

Archived normal ovarian and fimbrial tissues from premenopausal (<50 years old) and postmenopausal (56–85 years old) women, women with a BRCA1 mutation, as well as tumor tissues from women with ovarian HGSC were collected through an IRB-approved protocol from the Department of Pathology at Rush University Medical Center, Chicago, IL. Tumors were classified at the time of surgery. Tissues were examined with hematoxylin and eosin (H&E) staining. Normal ovaries (*n* = 30) and fimbria (*n* = 10) from pre- and postmenopausal women, BRCA1+ women (*n* = 10), and ovarian HGSC at early and late stages (*n* = 16) were selected for subsequent experiments based on H&E staining and final pathological reporting. A power analysis was performed to determine the minimum number of samples required to detect a true between-group difference of tissue and/or serum nucleolin levels at a statistical significance of α = 0.05 with power 1-β = 0.80. Blood samples from matched or unmatched subjects or patients were collected for Pathology immediately after they arrived for surgery. Sera were separated and aliquots were stored at −80 °C for later use.

### 2.2. Hen Specimens

All animal experiments including animal management and specimen collection were conducted under a protocol approved by the Institutional Animal Care and Use Committee (IACUC) of the University of Illinois Urbana-Champaign (UIUC). A flock of White Leghorn laying hens (4 years old) was reared under a standard husbandry practice in individual cages at the UIUC Poultry Research Farm. Hens were provided with layer ration and water *ad libitum* and egg-laying rates were recorded daily. Measures were taken to minimize pain or discomfort in all practices, including euthanasia via cervical dislocation or inhalation of carbon dioxide, consistent with the humane recommendations of the American Veterinary Medical Association.

### 2.3. Exploratory Study

From the flock, 240 hens with low egg-laying rates, had stopped egg-laying, or with distended water belly (an apparent sign of ascitic fluid) were scanned with TVUS as reported earlier [18]. Blood from all hens was collected from the brachial (wing) vein prior to euthanasia, serum was separated and stored at −80 °C for later use. Following euthanasia, hens were examined at gross for the presence of solid mass (tumor) and extent of the disease (metastasis, ascites), as reported earlier [21]. Normal or tumor-containing tissues including ovaries, oviductal fimbria, and other parts of the oviduct were harvested, processed, and stored for later use. Hens with detectable solid mass limited to the fimbria were grouped as *OVCA at an early stage* (*n* = 6). Hens with tumors that had metastasized to other organs in the peritoneal and pelvic cavities and were accompanied by profuse ascites were grouped as *OVCA at a late stage* (*n* = 6). In addition, hens with normal ovaries, normal oviductal fimbria, and no abnormality in any other organs were selected and grouped as *normal/healthy* (*n* = 8). The final diagnosis was made upon microscopic examinations, as reported earlier [21].

### 2.4. Prospective Study

From the flock, 210 apparently healthy hens without any detectable abnormality were selected by TVUS scanning. Hens were monitored for 60 weeks with TVUS scanning at 10-week intervals. Blood samples were collected at each scan and sera were separated and stored at −80 °C for later use. During the monitoring period, hens that developed a solid mass, as detected by TVUS, were euthanized once detected and tissues were collected, processed, and stored for later use. All remaining hens were euthanized at week 60 (at the end of the study) and tissues were collected, and processed for routine histology, immunohistochemistry, proteomics, and gene expression studies. The final diagnosis was made upon gross and microscopic examinations, as mentioned in the exploratory study and reported earlier [22,23].

### 2.5. Histopathology

Paraffin sections of 5µm thickness were made from formalin-fixed paraffin-embedded tissues and stained with H&E to confirm the presence or absence of malignancy and to classify histological types, when present, as reported earlier [17].

### 2.6. Immunohistochemistry

The cellular expression of nucleolin in patient and hen ovaries and fimbriae with or without HGSC tumor at all stages or in high-risk tissues was determined by immunohistochemistry using paraffin sections, as previously reported [21]. Sections were incubated with mouse anti-nucleolin (Millipore Sigma, St. Louis, MO, USA) or rabbit anti-collagen VI (Abcam, Boston, MA, USA) primary antibodies at a 1:100 dilution followed by biotinylated secondary antibodies and enzymes, and immune reactions were detected with 3, 3′Diaminobenzidine (DAB, Vector Laboratories, Burlingame, CA, USA). Sections were examined under a light microscope attached to a computer-assisted software program (MicroSuite^TM^ version 5, Olympus American, Inc., Center Valley, PA, USA), which was used to capture images and assess the intensity of the nucleolin staining. Intensities of immunohistochemical staining were measured as arbitrary values of pixels determined by the imaging software program mentioned above. The average intensities of nucleolin expression in all sections in a group (including normal or tumor) were considered as the mean intensity for the nucleolin expression in that group. Values are presented as the mean with the standard error of the mean (SEM) in a 20,000 μm^2^ area of the tissue. The specificity of nucleolin staining was confirmed by negative staining with the omission of the primary antibody.

### 2.7. Immunoblotting

Proteins from representative specimens from all tissue types were examined to determine the changes in nucleolin expression during OVCA development and progression, as reported earlier [21], using the primary antibody mentioned above, rabbit anti-actin (BioLegend, San Diego, CA, USA), or rabbit anti-PCNA (Abcam, Boston, MA, USA) antibodies at a 1:1000 dilution. Membranes were then incubated with sheep anti-mouse or goat anti-rabbit secondary antibodies conjugated with horseradish peroxidase. Immune reactions on the membrane were visualized with a chemiluminescent product (Millipore Sigma, St. Louis, MO, USA) under a ChemiDoc XRS (Bio-Rad Laboratories, Hercules, CA, USA). Images were captured and archived for later use. The signal intensity of each band was determined using the getIT! analysis software (Olympus Soft Imaging Solutions Corporation, Lakewood, CO, USA), as previously reported [24]. Signal intensities are presented as an intensity ratio (mean ± SEM).

### 2.8. Gene Expression Study

The expression of nucleolin gene *NCL* in clinical and preclinical normal and tumor tissues was examined using reverse-transcriptase polymerase chain reaction (RT-PCR) and quantitative RT-PCR (qRT-PCR) assays using actin as a loading control. Total mRNA extraction, cDNA synthesis, and qRT-PCR protocols were followed, as previously reported [21]. In qRT-PCR, the normal samples served as the baseline on which fold-change calculations were based. The following primers were used for the patient (*Homo sapiens*, *hs*) and hen (*Gallus gallus*, *gg*) samples (5′→3′):

Nucleolin (hs) F: CCCAGGGGATCACCTAATGC R: CTATCCTTGCCCGAACGGAG

Actin (hs) F: CCACCATGTACCCTGGCATT R: GTACTTGCGCTCAGGAGGAG

Nucleolin (gg) F: CACAACGGAGGAGACGCTAA R: GCATCTTCTGGGGAGCTGAA;

Actin (gg) F: TGGCAATGAGAGGTTCAGGT R: ATGCCAGGGTACATTGTGGT

### 2.9. Immunoassay

Immunoassays determined the circulating levels of nucleolin in serum samples from healthy subjects (*n* = 8), patients (*n* = 5 each for early and late stages), and hens (normal *n* = 8, early and late stages *n* = 6 each) using commercially available kits (MyBioSource, San Diego, CA, USA), as per the manufacturer’s recommendation. All standards (6 dilutions), blanks, and samples were plated in duplicate, plates were read at 450 nm, and the optical density (OD) of each sample was recorded. The mean OD value for each standard or experimental specimen was calculated. A standard curve was constructed by plotting OD values of standards against nucleolin concentration. The concentration of nucleolin in samples was determined with reference to the standard curve.

### 2.10. Cell Lines

Human ovarian surface epithelial (HOSE) cells and ovarian cancer cell line OVCAR3 (ATCC, Manassas, VA, USA) were cultured according to the manufacturer’s instructions using appropriate media. When confluent, cells were pelleted using a trypsin-EDTA solution (ThermoFisher Scientific, Waltham, MA, USA). A portion of the pellet was used to extract cytoplasmic and nuclear fractions using a commercial cell fractionation kit (Abcam, Boston, MA, USA), and the remaining pellet was processed as whole cell lysate. The efficiency of subcellular fractionation was confirmed by immunoblotting for proliferating cell nuclear antigen (PCNA), a nuclear marker indicative of proliferating cells, including tumor cells [25,26]. The concentration of protein was determined and lysates were used for immunoblots, as described above.

### 2.11. Statistical Analysis

All significant differences were determined by one-way analysis of variance (ANOVA), accounting for multiplicity using Tukey’s multiple comparison tests with a single pooled variance using GraphPad Prism Software (GraphPad Software Inc., La Jolla, CA, USA). Differences were considered significant when *P <* 0.05.

## 3. Results

### 3.1. Localization of Nucleolin Expression during Malignant Transformation Leading to Ovarian HGSC

**Figure 1:**
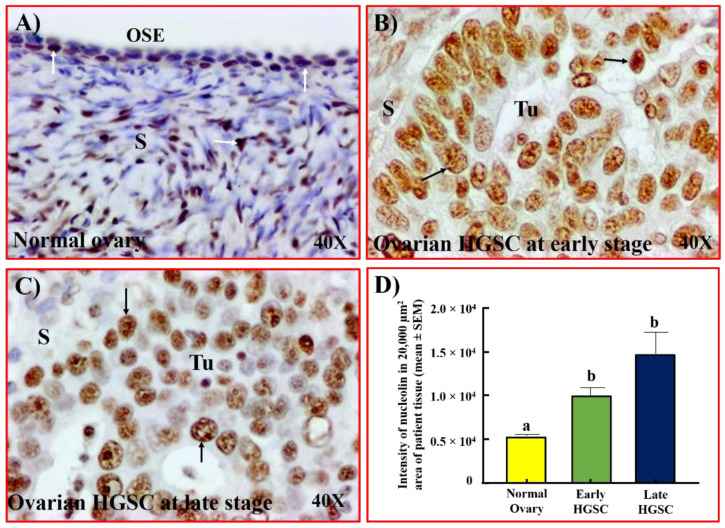
Localization of nucleolin in ovarian HGSC. (**A**) Section of a normal ovary showing the expression of nucleolin by a few OSE cells and stromal cells. (**B**,**C**) Sections of ovarian HGSC from patients with early (**B**) and late-stage (**C**) disease showing intense staining for nucleolin by malignant cells. (**D**) The intensity of the immunohistochemical staining of nucleolin was quantified and summarized as the mean ± SEM in a 20,000 µm^2^ area of tissue. Each bar represents the intensity of nucleolin expression in subjects with normal ovaries (*n* = 8) or patients with early (*n* = 5) and late stages (*n* = 5) of ovarian HGSC. Compared with normal ovaries, the expression of nucleolin was significantly higher in ovarian HGSC at early and late stages (*p* < 0.05). Bars with different letters are significantly different in their nucleolin expression (normal vs. early *p* < 0.05; normal vs. late *p* < 0.01). Arrows indicate examples of immunopositive cells. OSE = ovarian surface epithelium, S = stroma, Tu = tumor. Magnification = 40×.

Normal ovarian surface epithelium (OSE) showed weak expression for nucleolin with sporadic staining by the stromal cells (Figure 1A). In contrast, stronger immunoreactions for nucleolin expression were observed in malignant cells in ovarian HGSC at early and late stages (Figure 1 B,C). The intensity of the tissue expression of nucleolin in normal ovaries was 5.4 × 10^3^ ± 0.2 × 10^3^ in a 20,000 µm^2^ area of tissue. The intensity increased significantly to approximately 2-fold in patients with early (*p <* 0.05) and 3-fold in patients with late-stage (*p <* 0.01) ovarian HGSC (Figure 1D).

**Figure 2:**
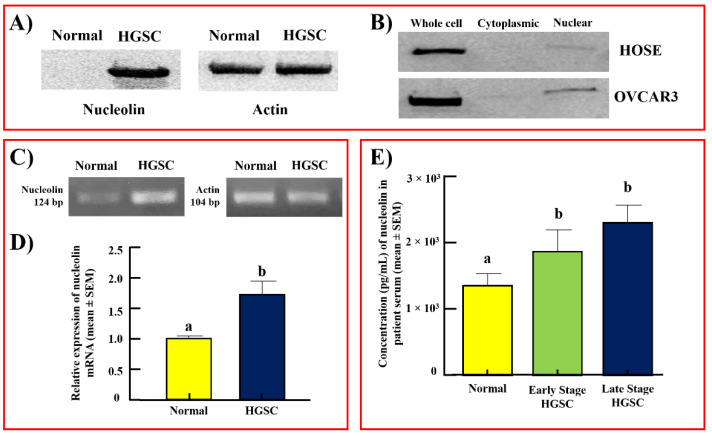
Changes in nucleolin expression during ovarian HGSC development as detected by immunoblotting, gene expression, and immunoassays. Immunoblot: (**A**) Immunoblot of tumor homogenate from an ovarian HGSC patient showing intense nucleolin expression while normal ovarian homogenate shows very faint expression for nucleolin. (**B**) Compared with HOSE (normal ovarian epithelial) cells, the immunoblot of whole cell lysate from OVCAR3 (ovarian cancer cell line) showed strong nucleolin expression. Similarly, a stronger signal for nucleolin was observed in the nuclear fraction from cancer cells (OVCAR3) than in normal ovarian (HOSE) cells. Gene expression assays: (**C**) RT-PCR assays showed stronger expression of *NCL* (nucleolin gene, 124 bp) in ovarian HGSC tumors than in normal ovaries. (**D**) Quantitative RT-PCR assay showing bars representing the expression of *NCL* in normal ovaries (*n* = 3) and ovarian HGSC (*n* = 5). Compared with normal ovaries, expression of the *NCL* gene was significantly higher in ovarian HGSC. Bars with different letters are significantly different in their expression of the *NCL* gene (*p* < 0.05). Immunoassays: (**E**) Bar graph representing the serum levels of nucleolin in subjects with normal ovaries (*n* = 8) and patients with early- (*n* = 5) and late-stage (*n* = 5) HGSC. Compared with normal subjects (1400 ± 105 pg/mL), the concentration of serum nucleolin was approximately 1.5- and 1.7-fold higher (*p* < 0.05) in the serum of patients with early- and late-stage ovarian HGSC, respectively. Bars with different letters are significantly different in their serum levels of nucleolin (normal vs. early *p* < 0.05; normal vs. late *p* < 0.05). Actin served as a loading control. Bp = base pair.

Immunoblotting detected nucleolin protein at a predicted molecular weight of 75 kDa in all samples with variable signal intensities. Normal ovaries yielded very weak (almost undetectable) reactivity, while ovarian tumors showed strong immunoreactive bands for nucleolin (Figure 2A). The observed immunoreactivity aligns with the results of the immunohistochemical staining. The immunoblot of whole cell lysates and subcellular fractions similarly detected nucleolin, with stronger reactivity observed in OVCAR3, an ovarian cancer cell line (Figure 2B). The efficiency of fractionation was confirmed by examining the expression of nuclear marker proliferating cell nuclear antigen (PCNA) in the immunoblot of subcellular fractions (Supplementary Figure S1). Furthermore, the signal intensity of bands appeared stronger in OVCAR3 nuclear fractions when compared to the nuclear fraction of normal human ovarian surface epithelial (HOSE) cells. Cytoplasmic fractions of both cell lines showed faint reactivity for nucleolin. The intensity ratios of the immunoblot signals support this result (Supplementary Figure S2). The cell line studies support the results observed in clinical specimens.

Semiquantitative RT-PCR showed stronger expression of the nucleolin gene (*NCL)* in ovarian HGSC than in normal ovaries (Figure 2C). Similarly, qRT-PCR showed a significant (*p* < 0.05) increase in *NCL* (approximately 1.75-fold) expression in ovarian HGSC compared with normal ovaries (Figure 2D). These results further confirmed the increase in nucleolin expression during the development of ovarian HGSC, as observed in the immunohistochemical and immunoblotting studies.

### 3.2. Serum Levels of Nucleolin

The immunoassay detected the prevalence of nucleolin in sera of women with or without OVCA. Nucleolin in the sera of healthy women was 1400 ± 105 pg/mL (mean ± SEM). Compared with healthy subjects, the serum level of nucleolin increased to approximately 1.5-fold in patients with early-stage ovarian HGSC (*p* < 0.05) and increased further in patients with late-stage disease (1.7-fold, Figure 2E). Thus, an increase in tissue expression of nucleolin during ovarian HGSC development and progression is accompanied by an increase in the serum levels of nucleolin.

### 3.3. Nucleolin Expression in Tissues with Risk of Ovarian HGSC

**Figure 3:**
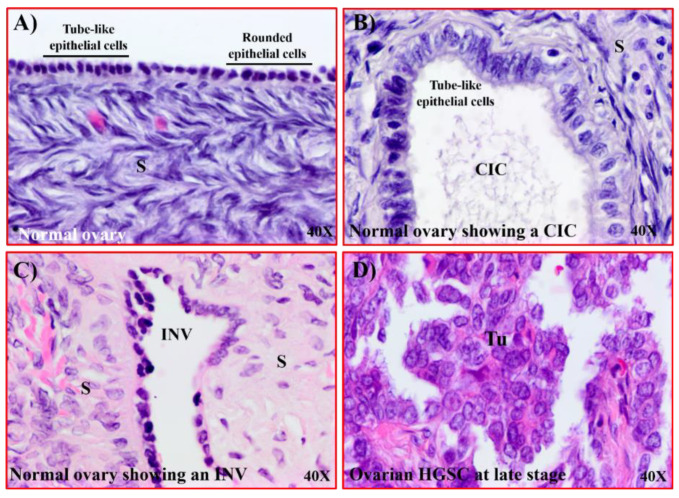
Microscopic features in ovaries with a risk of OVCA development. Sections were examined with H&E staining. (**A**) Section of a normal ovary showing both rounded and tube-like surface epithelial cells. (**B**) Section of a normal ovary showing a cortical inclusion cyst (CIC) embedded in the stroma containing a single layer of tube-like epithelial cells. (**C**) Section of a normal ovary showing a stromal invagination (INV) formed along the normal ovarian surface epithelium. (**D**) Section of an ovarian HGSC showing papillary structures and malignant cells containing pleomorphic nuclei. S = stroma, Tu = tumor. Magnification = 40×.

Following ovulation, OSE cells at the site of ovulatory rupture undergo morphological changes as part of their recovery from ovulatory injury leading to the formation of cortical inclusion cysts (CICs) and/or stromal invaginations (INVs) of the OSE. Though both CICs and INVs are formed from OSE cells, they are morphologically distinct features. The ovarian surface layer consists of rounded or columnar (tubular) epithelial cells or their mixture (Figure 3A). However, epithelial cells in CICs appear with a tube-like elongated morphology (Figure 3B), while epithelial cells in INVs are mainly rounded in shape (Figure 3C). The incidence and persistence of these microscopic features increase a woman’s risk of developing ovarian HGSC (Figure 3D).

**Figure 4:**
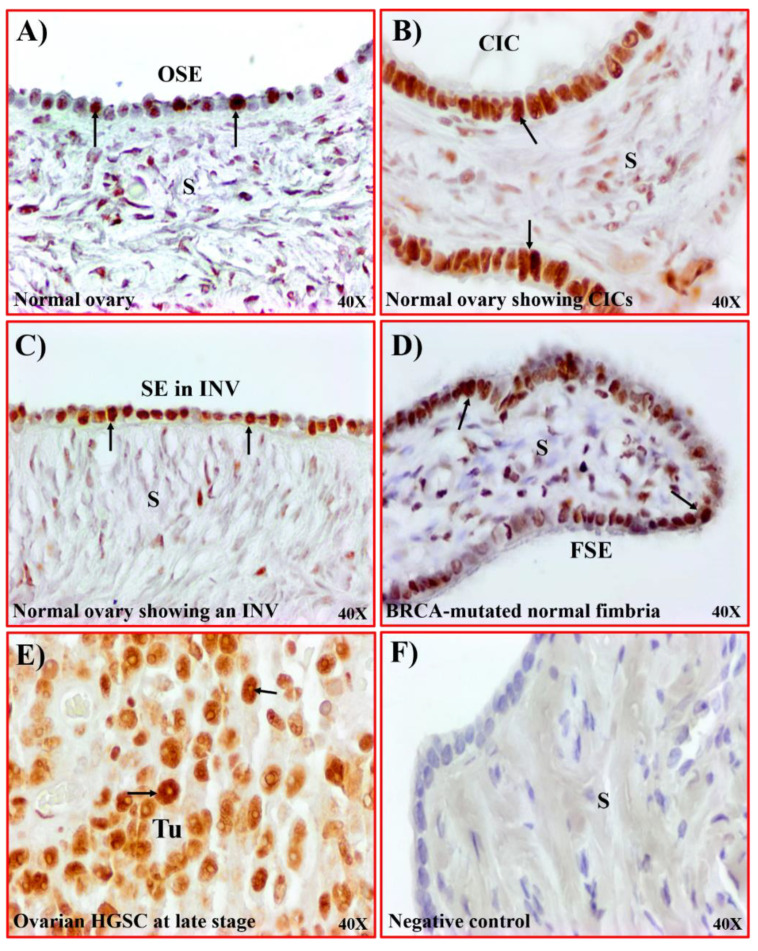
Expression of nucleolin by ovarian CICs and INVs. (**A**) Section of a normal ovary showing nucleolin expression by a few OSE cells. (**B**) Section of a normal ovary showing intense expression of nucleolin by the tube-like epithelial cells of the stromal CICs. (**C**) Section of a normal ovary showing nucleolin expression by the epithelial cells in a stromal invagination (INV). (**D**) Section of an oviductal fimbria from a BRCA+ woman showing nucleolin expression by the fimbrial surface epithelial (FSE) cells. (**E**) Section of an ovarian HGSC showing intense expression of nucleolin by the malignant cells. (**F**) Section of a normal ovary immunostained by omitting primary antibodies (negative control) in which no staining for nucleolin is observed. Arrows indicate a positive stain. S = stroma, SE = surface epithelium, Tu = tumor. Magnification = 40×.

As shown in Figure 1, a few OSE cells in the normal ovarian surface layer showed an expression for nucleolin (Figure 4A). In comparison, epithelial cells in CICs and INVs showed intense staining for nucleolin (Figure 4B,C). Furthermore, oviductal fimbria in women with BRCA mutation (BRCA+), a potential site of origin of HGSC, shows strong staining for nucleolin along the surface epithelium (Figure 4D). The expression of nucleolin in these high-risk tissues is comparable to the expression observed in ovarian HGSC (Figure 4E). No staining is observed in sections in which the primary antibody was omitted (Figure 4F). Paraffin sections of tumors were examined with collagen VI (a marker of stromal cells, [27]) for immunohistochemical staining of tumor stroma to confirm that nucleolin expression was limited to malignant cells (Supplementary Figure S3). Stromal cells showed immunostaining for collagen VI. Thus, the enhancement in nucleolin expression is associated with a high risk of OVCA development.

### 3.4. Nucleolin Expression in Hens

**Figure 5:**
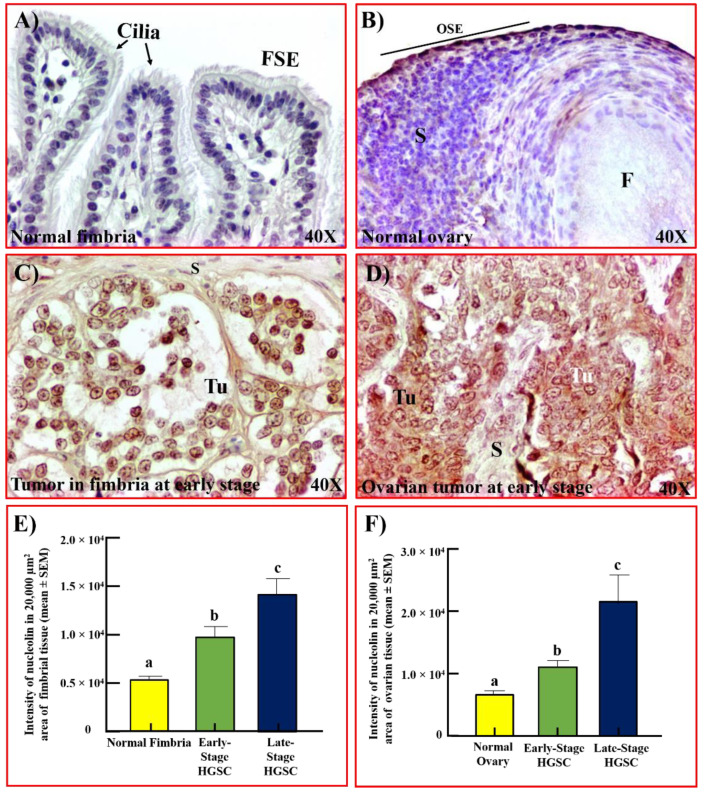
Nucleolin expression in normal and ovarian HGSC in hens. (**A**,**B**) Sections of normal oviductal fimbria (**A**) and ovary (**B**) in healthy hens showing very weak to moderate staining for nucleolin by the FSE and OSE cells, respectively. (**C**) Section of an oviductal fimbrial tumor showing strong expression for nucleolin. (**D**) Section of ovarian HGSC at an early stage in a hen showing staining for nucleolin. (**E**) The intensity of immunohistochemical staining of nucleolin was quantified and summarized as the mean ± SEM in a 20,000µm^2^ area of tissue. Each bar represents the intensity of nucleolin expression in hens with normal fimbria (*n* = 5) or fimbria with tumors at early (*n* = 6) and late stages (*n* = 4) of HGSC. Compared with normal fimbria, the intensity of nucleolin expression was significantly higher in fimbria with tumors at early-stage HGSC (*p* < 0.05) and in fimbria with tumors at late stages (*p* < 0.01). Bars with different letters are significantly different in their nucleolin expression. (**F**) The intensity of immunohistochemical expression of nucleolin in normal ovaries (*n* = 5) or ovarian HGSC at early (*n* = 5) and late stages (*n* = 5) summarized as the mean ± SEM in a 20,000µm^2^ area of tissue. Each bar represents the intensity of nucleolin expression in hens with normal ovaries or with ovarian HGSC at early and late stages. Compared with normal ovaries, the intensity of nucleolin expression was significantly higher in hens with ovarian HGSC at an early stage (*p* < 0.05) and increased further in hens with ovarian HGSC at late stages (*p* < 0.01). Bars with different letters are significantly different in their intensity of nucleolin expression. F = follicle, FSE/OSE = fimbrial/ovarian surface epithelium, S = stroma, Tu = tumor. Magnification = 40×.

A few surface epithelial cells in the oviductal fimbria of healthy hens showed very weak staining for nucleolin (Figure 5A). In normal ovaries, nucleolin expression in OSE cells was mainly localized in areas close to stromal follicles (Figure 5B). In contrast, strong staining for nucleolin was observed in tumors limited to the fimbria (Figure 5C) as well as in ovaries with HGSC (Figure 5D). The intensity of nucleolin was 5.4 × 10^3^ ± 0.3 × 10^3^ and 6.8 × 10^3^ ± 0.6 × 10^3^ in 20,000 µm^2^ in normal fimbriae and ovaries, respectively (Figure 5E,F). Compared with normal tissues, the intensity of nucleolin expression was approximately 2-fold greater in fimbriae with early-stage tumor (*p* < 0.05) and 1.6-fold greater in ovaries with early-stage tumor (*p* < 0.05). At late stages of ovarian HGSC, the intensity of nucleolin expression increased further to 2.6- (fimbria) and 3-fold (ovary) greater than normal (*p* < 0.05). 

**Figure 6:**
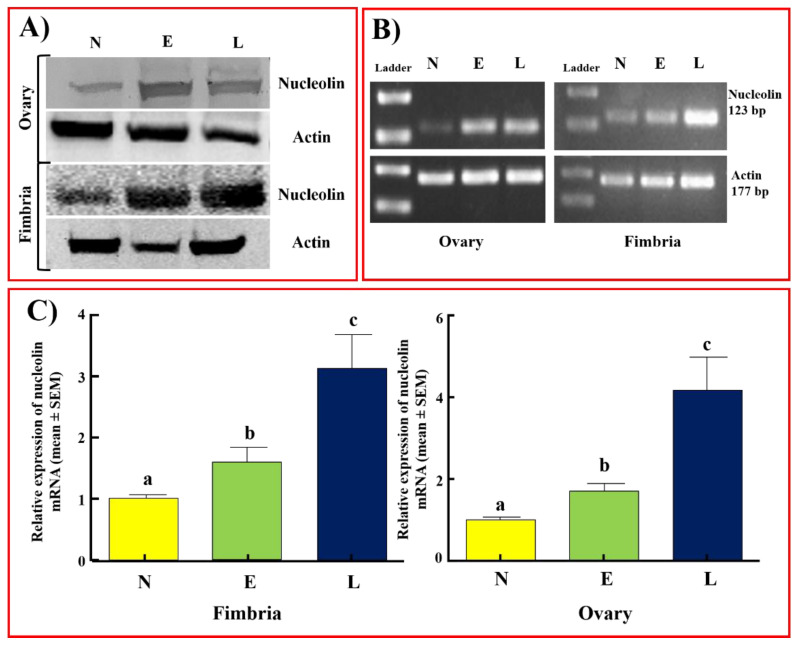
Changes in nucleolin expression during ovarian HGSC development in hens as detected by immunoblotting and gene expression assays. (**A**) Immunoblotting of ovarian or fimbrial tumor homogenates from hens with ovarian HGSC at early and late stages showed intense nucleolin expression, while normal ovarian or fimbrial homogenates showed weaker expression for nucleolin. (**B**) Semiquantitative (RT-PCR) gene expression assays showed stronger expression for *NCL* (nucleolin gene, 123 bp) in fimbrial or ovarian HGSC tumors than in normal ovary or fimbria. (**C**) Quantitative RT-PCR assays for *NCL* gene expression. Each bar represents the expression of the *NCL* gene in hens with normal ovaries (*n* = 4) or fimbriae (*n* = 4), and ovaries or fimbriae with tumors at early and late stages (*n* = 4 each) of HGSC. Compared with normal ovaries or fimbriae, the expression of the *NCL* gene was significantly higher (*p* < 0.01) in both ovarian and fimbrial HGSC at early stages and expression increased further in late stages (*p* < 0.001). Bars with different letters are significantly different in *NCL* gene expression (normal ovary vs. early-stage ovary *p* < 0.001; normal ovary vs. late-stage ovary *p* < 0.0001; normal fimbria vs. early-stage fimbria *p* < 0.01; normal fimbria vs. late-stage fimbria *p* < 0.001). Actin served as a loading control. Bp = base pair. E = early-stage HGSC, L = late-stage HGSC, N = normal.

Immunoblotting detected nucleolin protein of approximately 75 kDa in all samples examined with variable signal intensities (Figure 6A), in patterns similar to those observed in patients with ovarian HGSC. Compared to normal fimbriae and ovaries, tumors in fimbrial and ovarian HGSC at early and late stages displayed stronger immunoreactive bands for nucleolin. The intensity ratios of the immunoblot signals support this result (Supplementary Figure S2). The tumor-associated enhanced expression of nucleolin is consistent with immunohistochemical studies in hens. The expression of the *NCL* gene was also observed in all hen specimens. The expression of the *NCL* gene in RT-PCR was weak or faint in normal hens while it was stronger in fimbrial and ovarian tumors (Figure 6B). Quantitative gene expression (qRT-PCR) assays showed a significant increase in *NCL* gene expression in fimbrial tumors at early (1.45-fold, *p* < 0.01) and late stages (3.18-fold, *p* < 0.001) and in ovarian HGSC at early (1.54-fold, *p* < 0.05) and late (4.18-fold, *p* < 0.0001) stages when compared to normal fimbria and ovaries, respectively (Figure 6C). The observed changes in gene expression trends support the enhancement in nucleolin expression during the development of ovarian HGSC, as observed in the immunohistochemical and immunoblotting studies.

### 3.5. Serum Levels of Nucleolin in Hens

**Figure 7:**
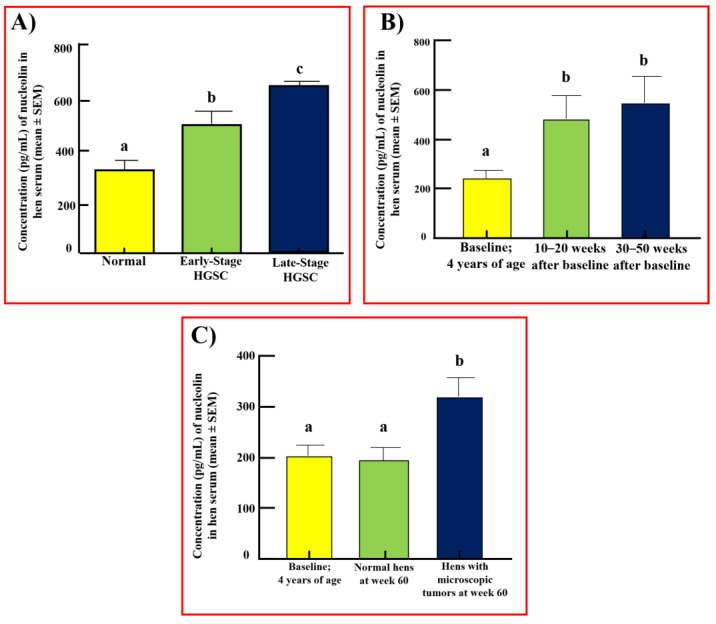
Changes in serum levels of nucleolin during HGSC development in hens. (**A**) Immunoassay showing serum levels of nucleolin (pg/mL, mean ± SEM) in hens with or without (*n* = 6) HGSC at early (*n* = 6) or late stages (*n* = 6). Each bar represents serum levels of nucleolin in normal hens or hens with HGSC at early and late stages. Compared with normal hens, nucleolin levels in serum were significantly higher in hens with ovarian HGSC at an early stage (*p* < 0.001) and increased further in late stages (*p* < 0.0001). Bars with different letters are significantly different in serum nucleolin levels (*p* < 0.001 for normal hens vs. hens with early-stage HGSC, and *p* < 0.0001 for normal hens vs. hens with late-stage HGSC). (**B**) Prospective study showing changes in serum levels of nucleolin during malignant transformation in hens that developed ovarian HGSC. Each bar represents serum levels of nucleolin in normal hens (baseline, immediately before monitoring), in hens at 10–20 weeks after baseline, and in hens at 30–50 weeks after baseline. Serum levels of nucleolin progressively increased in hens from baseline to 10–20 weeks of monitoring (*p* < 0.05) and further at weeks 30–50 of monitoring (*p* < 0.01) (baseline vs. 10–20 weeks after baseline *p* < 0.05; baseline vs. 30–50 weeks after baseline *p* < 0.01). (**C**) Hens that showed no apparent tumors at gross diagnosis were classified as normal or microscopic tumors at week 60 based on H&E staining. Healthy hens showed no change in nucleolin expression while hens with microscopic tumors showed an increase in serum levels of nucleolin (*p* < 0.05). Bars with different letters are significantly different in serum nucleolin levels, and bars with the same letter are not significantly different in serum nucleolin levels.

Exploratory Study: Compared with normal sera (312 ± 30 pg/mL), the serum level of nucleolin was significantly higher in hens with tumors limited to the fimbria or early-stage ovarian HGSC (507 ± 23 pg/mL) (*p* < 0.001, Figure 7A). The serum level of nucleolin increased further in hens with late-stage ovarian HGSC (660 ± 12 pg/mL, *p* < 0.0001). 

Prospective Study: During prospective monitoring, 12 hens developed solid mass (HGSC at an early stage) at 30 weeks of monitoring. In addition, seven hens at 40 weeks and eight hens at 50 weeks developed early-stage solid masses. The mean baseline (premonitoring) level of serum nucleolin in hens selected for monitoring was 217 ± 23 pg/mL (Figure 7B). The immunoassay showed that, compared with baseline (217 ± 23 pg/mL), serum levels of nucleolin were significantly higher (548 ± 91 pg/mL, *p* < 0.01) in hens that developed a detectable solid mass in weeks 30–50 of monitoring. The serum level of nucleolin in these hens showed an increase throughout the study period, including in weeks 10–20 of monitoring when no solid mass was detected (480 ± 85 pg/mL, *p* < 0.05). The tumor-associated enhanced levels of nucleolin in hens that developed a solid mass in the prospective study are comparable to the serum nucleolin levels in hens with early-stage ovarian HGSC observed in the exploratory study (presented in Figure 7A). Upon study completion, eight hens were found to have microscopic tumors not detectable by TVUS (Figure 7C). Hens with microscopic tumors showed an increase in serum nucleolin levels despite there being no solid mass (322 ± 32 pg/mL, *p* < 0.05). Hens that did not develop ovarian HGSC throughout the study period showed no significant change in serum nucleolin levels (Figure 7C).

## 4. Discussion

This study reports, for the first time, the enhancement in the expression of a nuclear protein, nucleolin, in patients with ovarian HGSC and in those with a high risk of developing this disease. This study showed that the tissue expression of nucleolin increases significantly during the development of ovarian HGSC. It also demonstrated that, in addition to patients with detectable ovarian malignant masses, the intensity of fimbrial and ovarian nucleolin expression was significantly higher in women with a high risk of OVCA development. Preclinical studies with hens, a model of spontaneous OVCA, showed that nucleolin becomes prevalent in the serum of hens even before a solid detectable mass is formed. Thus, changes in nucleolin expression are associated with premalignant changes in the fimbria and ovary leading to the development of ovarian HGSC.

As a nuclear protein, nucleolin plays a critical role in maintaining normal nuclear structure [28]. Nucleolin is also involved in a multitude of functions, including ribosome biosynthesis and chromatin remodeling [9], regulation of translation [29], and various signaling pathways [30,31]. Changes in the nuclear morphology of the cell, including irregular size and shape, are considered signs of malignant transformation and a harbinger of cancer [32]. The aberrant accumulation of nucleolin has been reported to be associated with several cancers including prostate [10], breast [11], glioblastomas [12], pancreatic [13], and hepatocellular carcinoma [14]. This study showed a significant increase in the tissue expression of nucleolin in ovarian HGSC at early and late stages, suggesting that changes in nucleolin expression are associated with OVCA development and progression.

In this study, compared to normal, the expression of nucleolin was significantly higher in epithelial cells in tissues with a risk of OVCA, including CICs and INVs (morphological features in ovaries formed by OSE cells after ovulatory rupture), and oviductal FSE cells in BRCA-mutated women. The reason(s) for increased nucleolin expression by the CICs and INVs are not known. CICs and INVs are sites of chronic inflammation with a risk of developing malignancy [24]. Chronic inflammation has been shown to be a hallmark of malignancy [33]. The enhanced expression of nucleolin was reported to be associated with inflammation [34,35], while the inhibition of nucleolin reduced inflammation [36]. Thus, our results suggest that nucleolin may be increased during chronic inflammation and premalignant changes in these tissues leading to OVCA development.

Following malignant transformation, transformed cells need to survive and proliferate to metastasize the tumor to other sites. In this study, immunohistochemical staining of tumor sections and sections of tissues from women with a risk of OVCA development, as well as immunoblotting of OVCAR3 cell lysates, showed the cell surface localization of nucleolin. Though the functions of cell surface nucleolin in OVCA are not known, cell surface nucleolin has been shown to prevent apoptosis by blocking the binding of Fas with FasL [37] and may be associated with cell proliferation through its interaction with ErbB1 and Ras oncoproteins [37]. Deregulated nucleolin expression has also been shown to promote cell migration and the propensity for metastasis via the activation of various pathways [38,39,40], while the inhibition of nucleolin has been shown to inhibit epithelial–mesenchymal transformation (EMT) [41]. In addition, nucleolin has been suggested to modulate the synthesis of matrix metalloproteinases, including MMP2, MMP7, and MMP9, facilitating the detachment of malignant cells and their migration [42]. This study showed that the intensities in the tissue expression of nucleolin increased during OVCA development and progression and thus future studies may examine the expression of nucleolin in metastatic sites. As observed in this study, the enhanced expression of nucleolin may be associated with OVCA development and progression, as reported by others for several cancers [40].

The rearrangements of nuclear materials and their organization are associated with an increase in the expression of several nuclear proteins and their shedding into circulation during malignant transformation [8,43]. The release of tumor-associated proteins into circulation offers an opportunity to detect malignant transformation or a growing tumor [44]. The exploratory part of this study with OVCA patients and the hen model for spontaneous OVCA showed the shedding of nucleolin in serum and its levels increased significantly during the development and progression of OVCA. These results suggest that serum nucleolin levels may predict OVCA development and progression. Furthermore, the enhanced expression of nucleolin in women with a risk of OVCA development, including BRCA+ subjects, suggests that nucleolin may be involved in the malignant transformation of these tissues. This assumption is also supported by the observations of the prospective study with hens that showed a significant increase in serum nucleolin levels even before a solid mass formed. This is further supported by the fact that apparently normal hens with microscopic tumors showed a significant increase in the serum level of nucleolin. Since healthy hens showed no significant change in the serum level of nucleolin throughout the study period, there are no time-associated changes in nucleolin that may confound these results. Thus, enhanced serum levels of nucleolin may be associated with malignant transformation of the fimbria and/or ovary, as well as OVCA progression.

The increased expression of nucleolin and its cell surface localization makes it a potential target for targeted therapy [45]. By inhibiting or blocking cell surface nucleolin, downstream targets associated with cell signaling pathways involved in survival, apoptosis-inhibition, cell proliferation, or cellular migration can be prevented to control tumor progression and recurrence. Nucleolin-targeted therapy in phase I and II clinical trials showed some success in renal cell carcinoma [46] and acute myeloid leukemia [47]. To the best of our knowledge, no clinical trial assessing nucleolin-targeted therapy in OVCA exists. Public databases [15] detail that nucleolin is expressed in all tissues and is increased in ovarian cancer, supporting the potential for nucleolin-targeted therapies to be effective in OVCA patients. Moreover, cell surface nucleolin can be targeted for molecular-targeted ultrasound imaging to improve the resolution and detectability of malignant-transformed cells or tissue for the detection of premalignant changes leading to fimbrial and/or ovarian tumors. The hen model of spontaneous OVCA is a feasible model for testing nucleolin-targeted therapies as well as monitoring the effectiveness of these targeted drugs. Using the hen model, we have developed ultrasound imaging agents targeting tumor-associated angiogenic microvessels [48,49] and ovarian inflammation-associated molecular markers of OVCA [50]. Thus, the hen model may also be used to develop and test nucleolin-targeted imaging agents for the detection of pre-metastatic early-stage OVCA.

The results of this pilot study showed the increased expression of nucleolin in ovarian and fimbrial HGSC as well as in tissues with a risk of ovarian HGSC development, including the fimbria of BRCA+ women, and in CICs and INVs in ovaries. An increase in tissue expression of nucleolin is accompanied by its shedding into circulation, the levels of which increase during malignant transformation, OVCA development, and progression. Although the sample size is smaller, the results of these studies will lay the foundation for a clinical study with larger cohorts.

## 5. Conclusions

In conclusion, the results of this study suggest that the tissue expression of nucleolin, a nuclear protein, and its serum levels increase during the development and progression of HGSC of the ovaries and fimbriae in patients and hens compared to healthy counterparts. The results of the prospective study suggest that nucleolin may predict malignant transformation prior to the formation of a solid mass.

## Data Availability

All data for this study is contained within the article and the Supplementary Materials.

## Supplementary Materials

The following supporting information can be downloaded at: https://www.mdpi.com/article/10.3390/cancers15030661/s1, Supplementary Figure S1. Uncropped and unedited Western blots from which final images were cropped for nucleolin, actin, and PCNA. In blots that include samples not pertinent to this manuscript, the bands of interest are outlined. Figure 2B—PCNA Upper Panel shows the full uncropped membrane of PCNA. Figure 2B—PCNA Bottom Panel shows a high-magnification and cropped version of the blot for clarity. W = whole cell; N = nuclear fraction; C = cytoplasmic fraction. Supplementary Figure S2. Intensity ratios of Western blot signals. Quantification graphs are shown for (A) blots from Figure 2A. (B) Blots from Figure 2B and Supplementary Figure 1. (C) Blots from Figure 6A. Ratios are presented as mean ± SEM from replicate readings. Supplementary Figure S3. Expression of collagen VI, a stromal cell marker, as determined by immunohistochemistry. (A) Staining for collagen VI in an early-stage ovarian HGSC shows immunopositive staining in the stromal cells between two sections of the tumor. No staining for malignant cells is observed. (B) Expression of collagen VI in late-stage ovarian HGSC similarly shows immunopositive staining for nonmalignant cells in the tumor stroma. Arrows indicate examples of positive stains. S = stroma; Tu = tumor. Magnification = 40X. Supplementary Figure S4. Uncropped and unedited RT-PCR gels from which final images were cropped for nucleolin and actin. In gels that include samples not pertinent to this manuscript, the bands of interest are outlined.
