# Supplementary material for: Changes in Nucleolin Expression during Malignant Transformation Leading to Ovarian High-Grade Serous Carcinoma
Source: Cancers (Basel). 2023 Jan 21;15(3):661. doi: 10.3390/cancers15030661 (PMC9913361; doi:10.3390/cancers15030661)
Supplement: Supplementary file 1 [file cancers-15-00661-s001.zip › cancers-2166156-supplementary.pdf]

Supplementary Figure S1. Uncropped & unedited Western blots

Figure 2A –Nucleolin

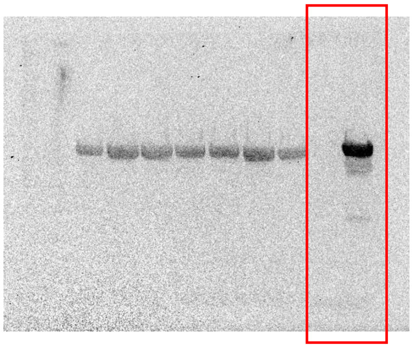

Figure 2B –Nucleolin

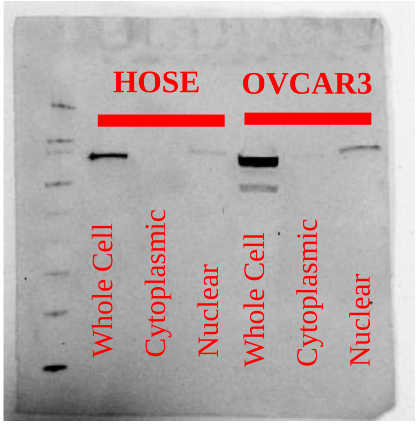

Figure 6A - Nucleolin

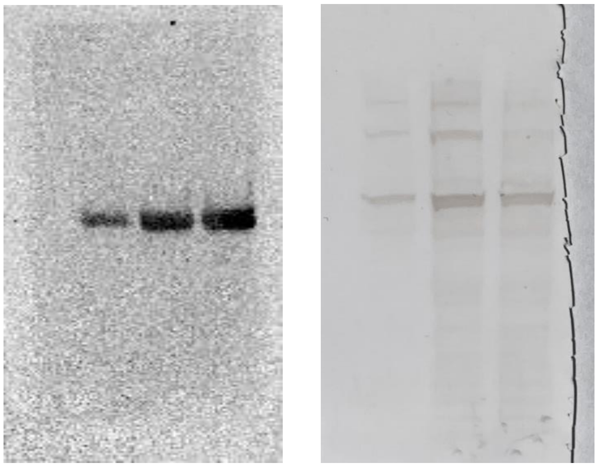

Fimbria

Ovary

Figure 2A – Actin

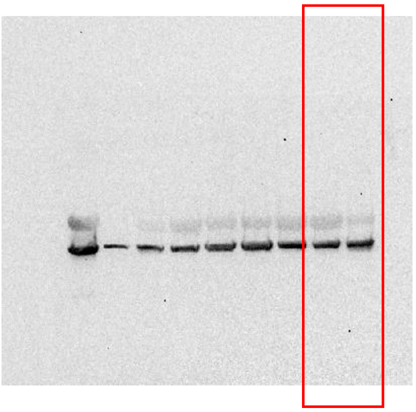

Figure 2B – PCNA

Upper:

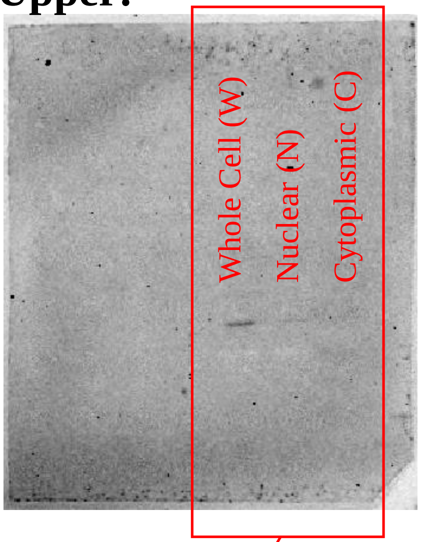

Bottom:

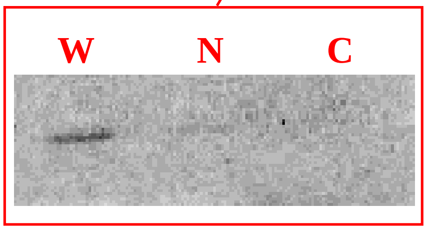

Figure 6A - Actin

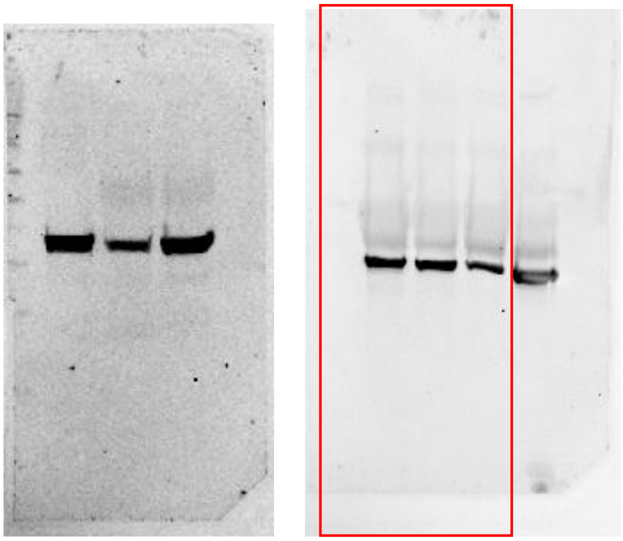

Fimbria

Ovary

Supplementary Figure S2. Intensity ratios of Western blot signals.

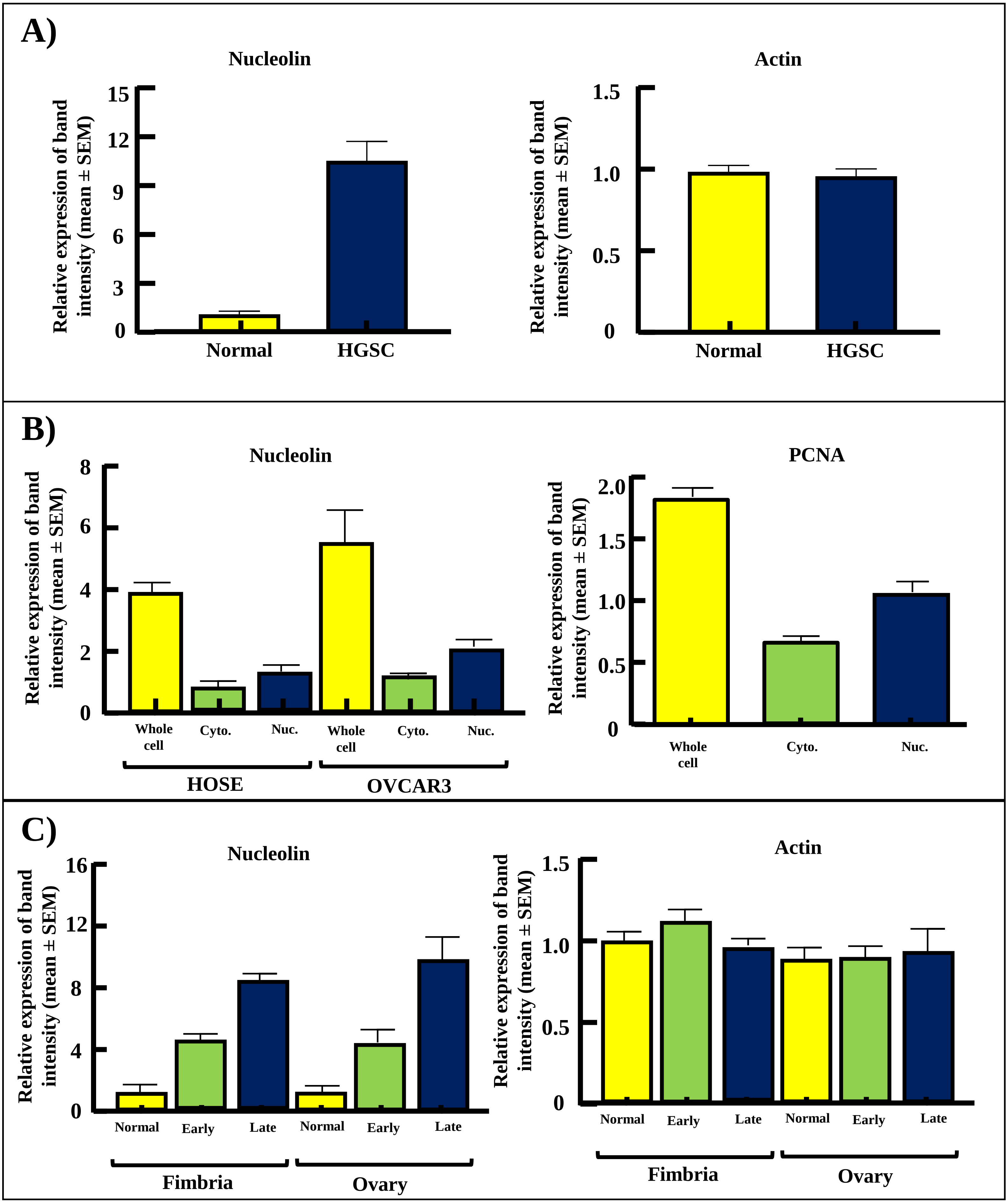

Supplementary Figure S3.
Immunostaining of Collagen VI, a stromal cell marker

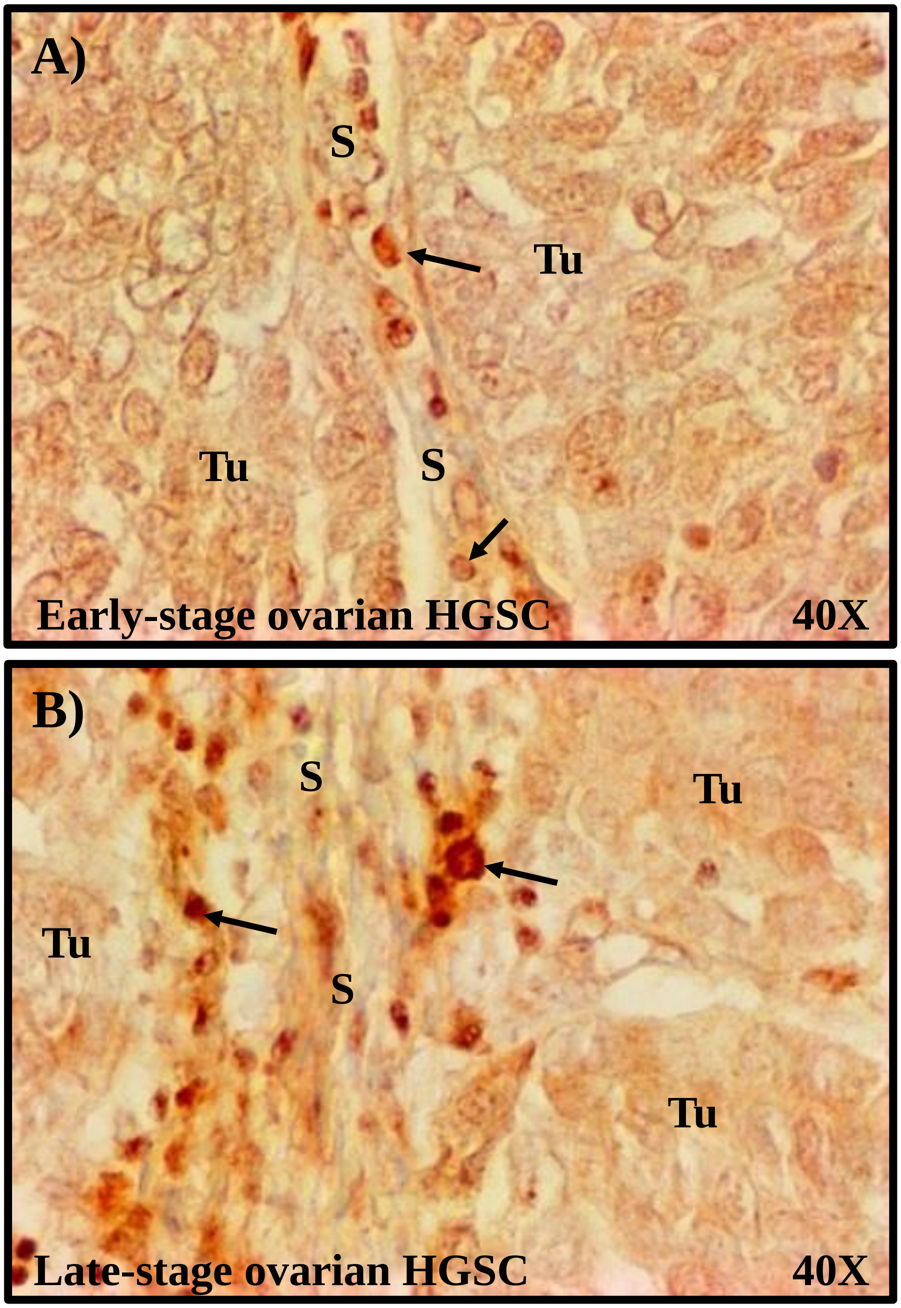

Supplementary Figure S4. Uncropped & unedited PCR gels

Figure 2C	Figure 6B - Fimbria	Figure 6B – Ovary
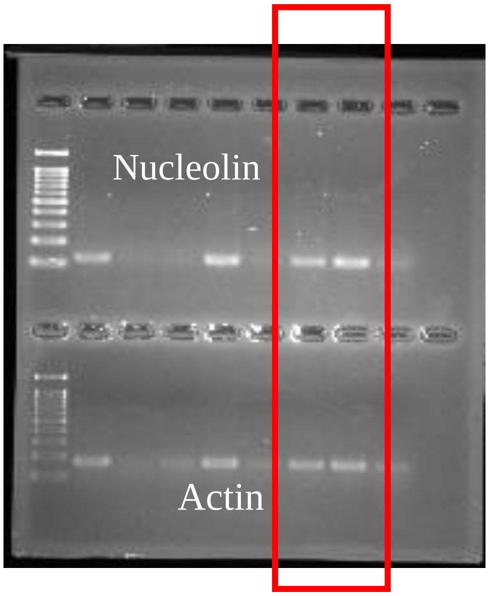	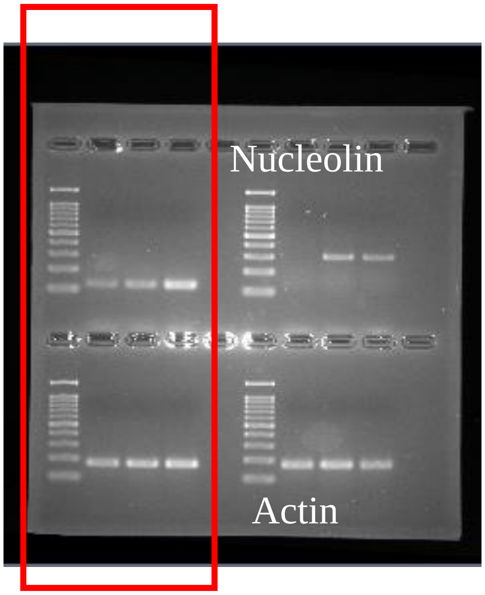	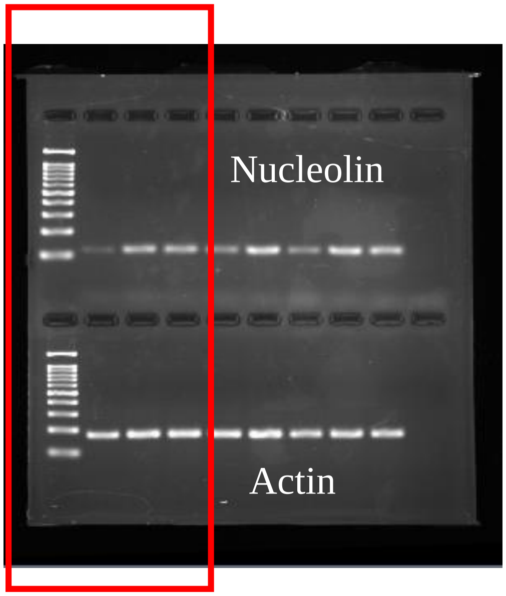
